# Childhood Allergies: The Role of Maternal Depression and Anxiety, and Family Strain

**DOI:** 10.3390/children8030185

**Published:** 2021-03-01

**Authors:** Ming Wai Wan, Molly Janta-Lipinski, Cemre Su Osam

**Affiliations:** 1 Perinatal Mental Health and Parenting Research Unit (PRIME-RU), School of Health Sciences, Faculty of Biology, Medicine and Health, University of Manchester, Manchester M13 9PL, UK; mollyjl597@gmail.com; 2 Centre for Women’s Mental Health, School of Health Sciences, Faculty of Biology, Medicine and Health, University of Manchester, Manchester M13 9PL, UK; cemresu.osam@manchester.ac.uk

**Keywords:** atopic disorder, asthma, eczema, postnatal depression, family relationships

## Abstract

Maternal mental disorder and a negative family emotional climate are a great source of stress for many children, yet their role in the childhood development or expression of asthma and allergies remains poorly understood, particularly beyond the first 1–2 years of life. The current study tested whether childhood allergy onset and symptomatology would be predicted by (1) perinatal and any time exposure to maternal depression or anxiety and (2) current family emotional strain (whole family, mother-child). UK mothers of children aged 2–12 years (N = 328) living with them completed an online survey of measures. Children exposed to maternal depression were almost twice as likely to be diagnosed and almost five times as likely to screen positive for an allergic disorder. Perinatal depression was linked to childhood allergies, but more moderately. Any anxiety exposure, and not specific to the perinatal period, predicted allergy status. Family emotional strain contributed independently to variance in concurrent child allergic symptomatology. All results were independent of potential confounders and current mental distress. The findings highlight the importance of maternal mental health and family function in the child’s neuro-immune development, and that these factors need to be addressed in the treatment of childhood allergic disorders.

## 1. Introduction

Allergic disorders are the most common chronic disease in Europe [1], are steadily increasing worldwide [2,3,4], and affect more than 20% of the population in most developed nations [5]. Common allergies (asthma, atopic dermatitis (eczema), and allergic rhinitis (e.g., hay fever)) arise from a heightened immune response and typically first present in childhood. Genetic susceptibility and exposure to environmental risk factors are understood to play a causal role [6,7]. Fetal and infant immune processes are sensitive to stress-induced biological changes, such as glucocorticoid exposure in utero, cytokine production, and/or fetal gastrointestinal microbiome imbalances [8,9]. Increasing animal and human research indicates that hypothalamic-pituitary-adrenal (HPA) function and other stress pathways may be altered epigenetically by stress in utero and in early caregiving experiences [10,11,12].

As much as a quarter of children living in the UK and elsewhere are exposed to maternal mental disorder [13,14], most commonly depression (18%) and anxiety (8%) [8]. Emerging evidence identifies maternal distress as a significant environmental contributor to the development of childhood allergies [15]. Children of perinatally distressed mothers are at raised risk of childhood asthma [16,17,18,19] and wheezing [12,20,21,22,23,24]. Risk of eczema increased with exposure to prenatal anxiety or depression [25,26]. Risk of rhinoconjunctivitis has been linked to prenatal maternal distress [27,28], and food allergy has been linked to perinatal stressful life events [29]. Beyond the perinatal period, a few studies have linked exposure to maternal distress at any time or cumulatively with raised child asthma risk [17,19], eczema [30], and altered child immune profiles [31,32]. Yet the focus on mental distress in the perinatal period may hide its true impact on child allergy risk, since maternal mental disorder at other times is similarly common [13] and tends to recur, and the immune system continues to develop through childhood with significant neuro-immune cross-talk [33]. Furthermore, many of these studies explicitly ruled out a reverse causation explanation, though health records of diagnosis or treatment are limited proxies for the timing of (allergy or mental disorder) onset.

A psychological or family systems approach, e.g., [34], recognises that maternal distress does not happen in a vacuum, nor does it necessarily reflect negative life events. Rather, maternal distress is inseparable from the family emotional climate (when, how often, and what types of emotions are expressed by household family members), and mother–child relationship qualities [35,36,37]. The mother’s mental health affects—and is affected by—family relationships that may amplify the child’s stressful environment, for example, by affecting how the mother interprets the child’s behaviour [38]. How adverse life events (which are perceived as stressful or not) are ‘absorbed’ by the family and impact on intra-familial relationships is critical in shaping the child’s perceptions of external threat, stress response, and thus potentially their developing immune system. While the role of a negative emotional family environment in allergy development has rarely been researched, a negative family climate has been shown to be linked to child asthma severity [37] and reduced expression of an inflammation downregulating glucocorticoid receptor gene in children with asthma [39]. On the other hand, positive family functioning and partner support appear to buffer the child’s genetic risk for asthma [16,36].

To gain a fuller understanding of the family environmental mechanisms that may contribute to the development of allergic disorders, the current study cross-sectionally investigated whether exposure to clinical levels of maternal mental depression and anxiety (from pregnancy to 12 years) and current family emotional strain are related to the childhood onset of allergies and allergic symptomatology scores. We tested two hypotheses: children exposed to maternal depression or anxiety, or a negative family climate (1) in the perinatal period (pregnancy to two years) or (2) at any time will significantly more likely have an allergy and more severe symptoms than those never exposed (or exposed more recently). We controlled for potential confounders: maternal history of allergic disorder (to help rule out a shared genetic pathway explanation [40]), key known environmental allergy causal factors (tobacco smoke exposure [41], antibiotics in pregnancy [42], and low socioeconomic status [43]), current mental distress (to help rule out self-report bias caused by current mental state) and the index (youngest) child’s age.

## 2. Materials and Methods

The participants were 328 English-speaking mothers residing in the UK who had (at least) one child aged 2–12 years living with them. Mothers were recruited by advertising widely online on parenting forums and social media to invite participation in a cross-sectional online survey study. The advertisement wording actively encouraged participation irrespective of the child’s allergy status. Following informed consent, participants completed several online measures anonymously. Multiparous mothers were instructed to complete the questions for their youngest eligible child (for consistency). All participants gave their informed consent for inclusion before they participated in the study. The study was conducted in accordance with the Declaration of Helsinki, and the protocol was approved by the University of Manchester Division of Psychology and Mental Health Research Ethics Committee (ref: 2019-8017-12621).

Maternal mental health and allergy-related factors: Multiple choice items asked about the mother’s self-reported lifetime depression and anxiety based on clinical diagnosis or prescription of medication, timing of such occurrence in relation to child exposure (pre-pregnancy, pregnancy, first year postpartum, second year postpartum, after the child’s second year), and perceived current management of mental health and stress (‘coping’; well overall, okay most days, not so well most days, not well at all most days, not well on any day). Information was also collated on mother’s self-reported allergy status, antibiotic use in pregnancy, and child tobacco smoke exposure (maternal or paternal/current partner smoking in pregnancy and currently). Mothers were asked whether the index child had been diagnosed with any allergies (asthma, atopic dermatitis (eczema), rhinitis (e.g., hayfever), and food allergy) and the age at which they presented.

Children Atopy Questionnaire (ChAt [44]): This 10-item parent-completed screener identifies child allergic disorder by asking about any diagnosis (1 item) and symptoms (9 items) associated with atopic disorders (all atopy is allergic). A screen-positive is based on a cut-off score of >2. The measure has high internal consistency (Cronbach α 0.76), high agreement with clinical diagnosis (based on clinical history, objective examination, and examination, including skin prick tests and specific Immunoglobulin E detection) (sensitivity = 92%; specificity = 90%), and identifies many who were previously undiagnosed in the general population (74.6% of positive screens [45]). After reviewing the English language items (original in Italian), the list of allergic disorders in item 1 (about diagnosis) was reduced to optimise comprehension (especially considering possible low health literacy in the UK) and because our focus was on asthma, atopic dermatitis (eczema), rhinitis (e.g., hay fever), and food allergy (with elimination diet). Specifically, hives and anaphylaxis, and the word ‘conjunctivitis’ (from the rhinitis description), were removed. Hay fever (e.g.,) was added in parentheses to clarify rhinitis. For scoring purposes, 1 point was assigned to item 1, irrespective of the number of diagnoses.

Hospital Anxiety and Depression Scale (HADS [46]): This 14-item measure is widely used to determine the levels of depression and anxiety an individual is experiencing. Depression and anxiety subscale scores are produced by asking about the cognitive and emotional aspects of mental health. HADS is designed to be self-administered and has a good internal consistency with a Cronbach α ranging 0.78–0.93 [47].

Family and child strain [48]: Questions on the mother’s perceived family emotional strain were selected as negative characteristics of the family emotional climate. Four items ask how often family members (living with the respondent) make emotional demands of them (i.e., make too many demands, get on your nerves, you feel critical of or criticised by, let you down) and 3 items about how often the index child (modified from the original that was on the partner) makes these same emotional demands on them (‘let you down’ was removed as not age appropriate, and the criticism item was extended to include ‘critical of’ as this was more parent–child relevant and to recognise the possible bidirectional nature of critical behaviour). Responses on a 4-point scale (not at all, rarely, sometimes, a lot) were totalled separately for family strain and child–mother strain. High scores equated to high emotional strain. Previous work reported adequate internal consistency (Cronbach’s alpha = 0.79).

Sociodemographic items included marital status, number of children, child age, child sex, mother’s ethnicity, mother’s educational level, and household annual income.

### Statistical Analysis

Firstly, descriptive statistics of the sample were produced. Childhood allergy was measured via two binary variables (diagnosis of at least one atopic disorder; ChAt screen positive status) and in terms of atopic severity (ChAt symptomatology score, after removing item 1 about diagnosis). We expect that allergy status based on diagnosis would be under-estimated, hence the inclusion of ChAt. Maternal depression and anxiety were determined by self-report of a medical diagnosis or (because formal diagnosis is rarely provided in primary care) prescription of medical treatment. The perinatal period was defined as from conception to two years postpartum. Low household income was defined as< GBP 18,000 per annum, which represents very low relative income, approximating the UK 10th percentile annualised net household income for a couple with 2 children <14 years of age, in 2017–2018 [49]. Exploratory chi square tests, Spearman correlations, and ANOVAs examined whether our childhood allergy variables (diagnosis, ChAt screen positive, atopic symptomatology) linked with sociodemographic characteristics, maternal mental distress, family strain, and established allergy-relevant factors. This also determined the predictors or potential confounders for included in subsequent analyses.

To address our hypotheses, logistic regression analyses quantified the association between exposure to maternal depression or anxiety (i) perinatally only, (ii) ever (i.e., at any time point including the prenatal period to the present time), and child allergic disorder and family strain, adjusting for maternal history of allergic disorder, prenatal antibiotic exposure, tobacco smoke exposure, indices of socioeconomic status, and current mental distress. Linear regression models tested the same predictors/confounders on child allergic symptomatology. Assumptions for the logistic and linear regression models were tested and met in preliminary analyses.

## 3. Results

### 3.1. Sociodemographic Factors by Child Allergy Status

Of the sample, 108 (32.9%) mothers described their (youngest) child as having been diagnosed with an allergy: asthma (*n* = 45; 14.7%), eczema (*n* = 63; 19.2%), allergic rhinitis (*n* = 19; 5.8%), and/or food allergy (*n* = 41; 12.5%). A larger proportion (N = 132; 40.2%) screened positive on the ChAt. Known allergy-related factors which we found to be more common among allergy-diagnosed children (prenatal antibiotics, tobacco in household, maternal allergy, non-university educational level) (Table 1) were controlled for in the main analyses, while low household income was not. No age by allergy diagnosis effect was found, but we controlled for child age, as older children had a longer time span for mental disorder [8] and potential allergy-related exposures. Allergy status based on the ChAt showed a similar pattern of effects, except that tobacco exposure did not reach significance. As clinical measures were based on maternal report, main analyses also adjusted for current mental health (HADS depression and anxiety scores), despite no group effects (Table 1).

### 3.2. Maternal Mental Health and Family Strain

Of 328 children, N = 38 (11.6%) had been exposed to maternal depression and N = 43 (13.1%) to maternal anxiety (perinatally: N = 32; 9.8% and N = 21; 6.4% respectively). Depression and anxiety frequently co-occurred (perinatal: X = 14.17; *p* < 0.001; any exposure: X = 94.51; *p* < 0.001). Thus, 63.2% of mothers with depression at any time since pregnancy had also been anxious. As past research suggested that differential depression and anxiety links with child allergy onset [50], and each disorder may impact the child’s biology differently [51,52], the main analyses were performed for depression and anxiety separately.

Children who had received an allergy diagnosis were more likely to have been exposed to clinical depression during their lifetime and prenatally, though this was a non-significant trend when depression status was based on the mother’s lifetime (Table 1). The pattern of results was reversed for maternal anxiety; children with an allergy diagnosis were more likely to have a mother with clinical anxiety in her lifetime. Mothers of the allergy diagnosis group also reported poorer current coping (management of mental health and stress) compared with the no allergy diagnosis group. On the other hand, ChAt screen positives were much more likely to have been exposed to clinical depression during their lifetime and prenatally, but not any more likely than the screen negatives to have had clinical anxiety. Neither were there screen-positive differences in current coping. Current maternal depression, anxiety, and family strain scores did not differ between groups, regardless of whether diagnosis or screen positive was used to measure allergy status, except for a trend in slightly raised family strain scores in the allergy group.

In an attempt to exclude the possibility that child allergy (and its management) is causing maternal distress, we examined (current) depression and anxiety scores among mothers whose child screened positive for an allergic disorder and found no differences compared with mothers whose child screened negative (depression: F = 1.15; *p* = 0.29; anxiety: F = 0.08; *p* = 0.78).

### 3.3. Child Allergy Symptom Severity

The ChAt was also used as a measure of allergic symptomatology (i.e., range of symptoms) once the first item (on diagnosis) was removed from the score. Children had more symptomatology when their mother had an allergy or had taken antibiotics prenatally (Table 2). This score was also modestly associated with family strain and not child strain. As child strain was not associated with any allergy outcomes, this was excluded from the main analyses. While allergy symptomatology was not linked with current depression or anxiety scores, symptom scores were higher with maternal depression and anxiety exposure, especially perinatal depression.

### 3.4. Testing Our Hypotheses: A Role of Maternal Mental Distress and Family Strain in Childhood Allergy

In the logistic regression models of childhood allergy containing depression as predictors (Models 1, 2, 5 and 6; Table 3), both any and perinatal depression independently predicted the presence of childhood allergy based on both outcome measures (diagnosis, ChAt screen positive status). Children were almost twice as likely to be diagnosed with an allergic disorder (OR: 1.98; CI: 1.02–3.82) and almost five times more likely to screen positive on the ChAt (OR: 4.96; CI: 2.10–11.67) if exposed to maternal depression at any time. If the mother experienced perinatal depression, children were more than twice as likely to be diagnosed with an allergic disorder (OR: 2.33, 95%; CI: 1.00–5.41) and almost four times as likely to screen positive (OR: 3.70; 95% CI: 1.56–8.80). Family strain had a significant modest independent predictive effect on a screen positive status and not allergy diagnosis (Model 5 and 6).

In the logistic regressions focused on anxiety (Models 3, 4, 7 and 8; Table 3), anxiety exposure at any time but not specific to the perinatal period predicted allergy status (based on diagnosis and ChAt screen positive). Children were over twice as likely to be diagnosed with an allergic disorder (OR: 2.13; 95% CI: 1.02–4.47) and to screen positive on the ChAt (OR: 2.44; 95% CI: 1.16–5.14) if exposed to maternal anxiety at any time. Current anxiety score was controlled for and appeared to exert an effect on screen positive status. Family strain did not account for any significant variance in these models.

In the four linear regression models of current allergic symptomatology (Table 4), exposure to depression perinatally or at any time and anxiety at any time contributed to the variance in symptomatology. Perinatal anxiety did not (Model 3). Family strain was also a significant predictor in all models, making a modest independent contribution to all models.

## 4. Discussion

While previous studies focused mainly on maternal distress in the perinatal period [16,17,18,19,20,21,22,23,24,25,26,27,28,29], the current findings support the role of maternal mental disorder not limited to the perinatal period in the development of childhood allergies, and the role of the family emotional climate in the expression of allergic symptoms. This study of UK mothers of children aged 2–12 years found that maternal depression and anxiety were associated with an increased risk of childhood allergies (asthma, eczema, rhinitis, food allergy), independent of the effects of other known allergy risk factors, current mental health, maternal education, and child age. In support of a family systems model, maternal depression exposure at any time was linked with increased odds of having a child screen positive for allergic disorder by almost five times. Exposure to maternal anxiety at any time also predicted childhood allergy and a range of allergic symptoms, albeit less strongly, doubling the odds of screening positively. Exposure to maternal depression or anxiety and family emotional strain are likely to be stressful to children in multiple ways (e.g., through affecting parenting style and emotional availability) which impacts on immune function via multiple mechanisms, such as alterations in glucocorticoids and cytokines. Our findings demonstrate the need to address maternal mental health difficulties and family strain, which are amenable to intervention through family systemic psychotherapy [34] or individual treatment.

We hypothesised that the perinatal period was a sensitive period when immune system development is susceptible to stress-related disruption. By contrast, weaker and no effects were found when focused on the perinatal period based on depression and anxiety exposure, respectively. However, most of the clinical depression subsample (89%) had experienced depression perinatally (exclusively or as well as other time points). Thus, the reduced power of the small perinatal-only subsample will have contributed to the lack of perinatal effects. Compared with UK primary care data, we report a lower rate of maternal depression at any time (12% vs. 18%) [13]. Relatively few women (*n* = 21; 6.4%) were diagnosed or medically treated for anxiety during the perinatal period. However, this is comparable to the prevalence rate reported for the perinatal period (6.5%). While universal antenatal care and a community health visiting programme in the UK place an emphasis on the mother’s mental health, depression and anxiety continue to go undiagnosed. The use of retrospective measurement of mental distress based on diagnosis or treatment will have underestimated mental distress exposure that can only be captured with prospective longitudinal study.

Family emotional strain was moderately related to the mother’s report of child atopic symptomatology, independent of current depression and anxiety scores, clinical history, and maternal education. This finding might suggest that an emotionally stressful family environment, independent of the impact of maternal mental health, may be a precipitating factor in atopic expression or may challenge the effective management of symptoms. By contrast, mother–child emotional strain was not linked to any of our allergy outcomes, suggesting that the broader family emotional climate is relevant rather than specific mother-to-child effects. Further work to identify specific aspects of family dynamics that lead to family emotional strain, such as partner (inter-parental) conflict and family communication styles (e.g., emotional invalidation), especially in dealing with negative life events and daily hassles, could help guide intervention development.

Furthermore, the study provides support for the use of the ChAt as a screening tool in a UK sample. A screen positive result was related to allergy-related factors in expected ways and generally more strongly with maternal mental health than they related to mother-reported child allergy diagnosis. The latter point is as expected given that allergies commonly go undiagnosed. Our exploratory analyses suggest that mothers who took antibiotics in pregnancy and mothers who were non-university graduates may be more motivated to seek diagnosis (and treatment) for their child. However, the higher anxiety scores of mothers of ChAt screen positive children (but not of allergy-diagnosed children) could suggest that reporting is confounded by current anxiety or that currently anxious mothers are more vigilant about their child’s health (but do not necessarily seek a medical diagnosis or treatment).

While childhood allergies (and their management) may cause maternal stress [53], several findings may place the weight of evidence against a reverse causation explanation for the current results. Firstly, while mothers of children who screened positive for allergic disorder (versus screen negatives) reported more difficulty in managing their stress levels and mental health, they did not report more depression or anxiety symptoms per se (based on current scores). Maternal depression and anxiety are related to but not synonymous with stress. Secondly, current depression and anxiety scores were controlled for in the main analyses, so the raised risk of child allergies among mothers diagnosed with depression or anxiety was irrespective of mothers’ current depressive or anxious symptoms. Thirdly, the mother–child relationship may be more strained if the child’s allergies compromised maternal mental health, but we found no group differences in mother–child emotional strain. Finally, that 89% of the clinically depressed women in our sample had been depressed prenatally (exclusively or as well as other times) suggests that child allergies are not the main cause of clinical depression or anxiety. The child’s inheritance of a strong proinflammatory response underlying both immune reactivity and stress susceptibility is more difficult to rule out, though we did control for maternal allergies and current mental distress.

Other limitations need to be acknowledged. As a cross-sectional study, measures relied on maternal recall of child allergy information and maternal mental health history. However, the screening measure complemented maternal reports of diagnosis and treatment. Secondly, to reduce the time burden of completing the survey, no measure of paternal mental health or partner conflict was taken. Paternal allergy information was lost or missing due to a clerical error on the survey web host, so the mother’s allergy status provided a partial measure of genetic allergic propensity. Previous work suggests that paternal mental distress is minimally influential on child allergy development [19,30]. Thirdly, our sample size was a limitation in testing the perinatal hypothesis and prevented us from investigating the effects of maternal distress on different types of allergies. Fourthly, we did not specify that participants had to be biological mothers. While a small proportion of non-biological or non-birth mothers consented to participate, it is likely that they would have withdrawn from participation partway through due to the inapplicability of several questions (e.g., perinatal mental health, prenatal antibiotic usage). The study is likely to have attracted mothers with concerns about their child’s allergies: most mothers (91%) reported having an allergy and the rate of child food allergy was high (12.5%), so these children may have had high genetic susceptibility, possibly leading to underestimation of environmental (e.g., maternal distress) effects. Further, non-White mothers were highly under-represented (6%) in this study.

Pending replication in a larger sample, the findings implicate the need to directly address parental mental health and family function as an integral part of treating childhood allergic disorders [34,54]. From the child’s perspective, acknowledging in discussion with families the potential stress and impact that child illness has on the family opens up the opportunity for sensitive inquiry around possible chronic family relational and external stressors, including parental mental health, which may emotionally stress the child or make allergy management challenging to parents [34]. Regarding implications for the parent, the findings highlight the importance of treating maternal mental health, not only to optimise the child’s social-emotional development and family functioning, but also due to the fact that stress pathways may be closely linked with immune system development, especially in cases of genetic allergic susceptibility. Future studies with larger samples and prospective data on timing of onset could better understand the links between maternal mental health and family emotional climate, and different patterns of allergic expression among children. As a test of causal pathways, future trials involving maternal mental health interventions, especially those that focus on family-based approaches, could also evaluate childhood allergy outcomes.

## Figures and Tables

**Table 1:** Child allergy group differences by allergy-related, family/clinical, and sociodemographic variables: descriptive statistics, chi square tests, and analyses of variance (N = 328).

Allergy Diagnosis *n* (%)/M (SD)				ChAt Screen Positive *n* (%)/M (SD)		
	Yes N = 108	No N = 220	Group Difference	Yes N = 132	No N = 196	Group Difference
			X			X
Low income	20 (18.5%)	33 (15.0%)	0.66	23 (43.4%)	30 (15.3%)	0.26
M non-graduate M graduate	66 (61.1%) 42 (38.9%)	94 (42.7%) 126 (57.3%)	9.80 **	75 (56.8%) 57 (43.2%)	85 (43.4.3%) 111 (56.6%)	7.32 *
Index child male sex	58 (53.7%)	117 (53.2%)	0.01	72 (54.5%)	103 (52.6%)	0.13
M non-White ethnicity	6 (5.6%)	15 (6.8%)	0.19	6 (4.5%)	15 (7.7%)	1.27
Tobacco exposure	56 (51.9%)	84 (38.2%)	5.53 *	63 (47.7%)	77 (39.3%)	2.30
Prenatal antibiotics	33 (30.6%)	31 (14.1%)	12.50 ***	35 (54.7%)	29 (14.8%)	6.90 **
M allergy status	79 (73.1%)	128 (58.2%)	6.97 **	99 (75.0%)	108 (55.1%)	13.41 ***
			*F*			*F*
Index child age	4.72 (2.68)	4.90 (2.80)	0.29	4.55 (2.61)	5.04 (2.84)	2.51
Depression score	5.62 (3.73)	5.15 (3.68)	1.19	5.57 (3.76)	5.12 (3.66)	1.15
Anxiety score	8.51 (4.33)	8.00 (4.24)	1.01	8.25 (4.29)	8.12 (4.26)	0.08
Family strain	6.42 (2.59)	5.86 (2.84)	2.91 ^+^	6.37 (2.67)	5.83 (2.82)	3.07 ^+^
M-child strain	4.07 (2.03)	4.10 (2.24)	0.01	4.01 (2.01)	4.15 (2.27)	0.35
M coping	4.05 (0.89)	4.26 (0.70)	5.51 *	4.11 (0.85)	4.24 (0.72)	2.10
			X			X
M clinical depression						
Perinatal	16 (14.8%)	16 (7.3%)	4.68 *	20 (15.22%)	12 (6.1%)	7.30 **
C exposure	18 (16.7%)	20 (9.1%)	4.06 *	24 (18.2%)	14 (7.1%)	9.38 **
M ever	23 (21.3%) ^+^	40 (18.2%)	0.45	33 (25.0%)	30 (15.3%)	4.78 *
M clinical anxiety						
Perinatal	9 (8.3%)	12 (5.5%)	1.00	10 (7.6%)	11 (5.6%)	0.51
C exposure	19 (17.6%) ^+^	24 (10.9%)	2.84	22 (16.7)	21 (10.5)	2.45
M ever	33 (30.6%)	40 (18.2%)	6.41 *	35 (26.5%)	38 (19.4%)	2.32

^+^*p* < 0.1; * *p* < 0.05; ** *p* < 0.01; *** *p* < 0.001; M = maternal; C = child.

**Table 2:** Child allergy symptomatology in relation to maternal mental health, family strain, and sociodemographic and health characteristics: ANOVA and correlations (N = 328).

Presence of Factor	Yes		No		F Value
	N	Mean (SD)	N	Mean (SD)	
Below graduate education	160	2.26 (2.08)	168	1.96 (2.03)	1.66
Low income	53	2.17 (1.95)	275	2.09 (2.08)	0.06
M non-White ethnicity	21	2.47 (2.42)	307	1.95 (2.13)	1.03
Tobacco exposure	145	2.28 (2.06)	183	1.97 (2.04)	1.77
Prenatal antibiotics	64	2.84 (2.12)	264	1.93 (2.00)	10.52 ***
M allergy	207	2.44 (2.17)	121	1.53 (1.71)	15.84 ***
M clinical depression					
Perinatal	32	3.19 (2.36)	296	1.99 (1.99)	10.10 **
C exposure	38	2.73 (2.27)	290	1.96 (1.98)	7.31 **
M ever	63	3.10 (2.63)	265	2.28 (2.33)	5.93 *
M clinical anxiety					
Perinatal	21	2.38 (2.38)	307	2.09 (2.03)	0.40
C exposure	38	3.37 (2.44)	290	1.94 (1.94)	16.99 ***
M ever	73	2.42 (2.24)	255	2.02 (1.99)	2.26
ChAt symptomatology (Spearman correlation r value)					
Index child age	Depression score	Anxiety score	Family strain	M-child strain	M coping
−0.07	0.06	0.01	0.13 *	0.01	−0.05

* *p* < 0.05; ** *p* < 0.01; *** *p* < 0.001; M = maternal; C = child.

**Table 3:** Logistic regression models of exposure to maternal distress (perinatal and any time) and family strain adjusted for potential confounders.

	Child Allergy Diagnosis					ChAT Screen Positive			
Model	1: PD	2: AMD	3: PA	4: AMA	5: PD	6: AMD	7: PA	8: AMA
	OR (95% CI)				OR (95% CI)			
M education	1.72 (0.99–2.99) ^+^	1.71 (0.98–2.98) ^+^	1.66 (0.95–2.87) ^+^	1.67 (0.96–2.91) ^+^	1.57 (0.91–2.68)	1.62 (0.94–2.79)	1.49 (0.87–2.53)	1.50 (0.88–2.57)
Tobacco exposure	1.47 (0.85–2.54)	1.47 (0.85–2.55)	1.49 (0.87–2.57)	1.47 (0.85–2.54)	1.24 (0.73–2.11)	1.21 (0.71–2.08)	1.26 (0.75–2.14)	1.24 (0.73–2.11)
Prenatal antibiotics	2.35 (1.31–4.23) **	2.39 (1.33–4.31) **	2.43 (1.36–4.37) **	2.56 (1.42–4.61) **	1.80 (1.00–3.23)	1.88 (1.04–3.39)	1.89 (1.06–3.37) *	1.99 (1.11–3.58) *
M any allergy	1.86 (1.09–3.18) *	1.81 (1.06–3.09) *	1.82 (1.07–3.11) *	1.95 (1.13–3.36) *	2.42 (1.44–4.07) ***	2.44 (1.44–4.11) ***	2.32 (1.39–3.87) ***	2.51 (1.49–4.24) **
Index child age	0.98 (0.89–1.07)	0.97 (0.88–1.06)	0.99 (0.90–1.08)	0.98 (0.89–1.07)	0.93 (0.85–1.02)	0.91 (0.84–0.98) *	0.95 (0.87–1.03)	0.93 (0.85–1.02)
Depression score	0.98 (0.89–1.07)	0.97 (0.89–1.06)	0.99 (0.91–1.09)	1.00 (0.91–1.09)	0.92 (0.85–0.99)	0.90 (0.82–0.99)	1.01 (0.93–1.10)	1.02 (0.94–1.11)
Anxiety score	0.96 (0.89–1.04)	0.96 (0.89–1.03)	0.97 (0.90–1.05)	0.95 (0.88–1.03)	0.99 (0.91–1.09) *	0.98 (0.90–1.07) *	0.94 (0.87–1.01) ^+^	0.92 (0.85–0.99)
Family strain	1.08 (0.98–1.19)	1.08 (0.98–1.19)	1.06 (0.96–1.17)	1.07 (0.97–1.18)	1.10 (1.00–1.21)	1.11 (1.01–1.23) *	1.08 (0.98–1.19)	1.08 (0.99–1.19) ^+^
PD	2.33 (1.00–5.41) *	-	-	-	3.70 (1.56–8.80) **	-	-	-
AMD		1.98 (1.02–3.82) *	-	-	-	4.96 (2.10–11.67) ***	--	-
PA	-	-	1.33 (0.51–3.50)	-	-	-	1.22 (0.61–2.43)	-
AMA	-	-	-	2.13 (1.02–4.47) *	-	-	-	2.44 (1.16–5.14) *

CI, confidence interval; PD, perinatal depression; AMD, any maternal depression; PA, perinatal anxiety; AMA, any maternal anxiety; M, maternal. ^+^ *p* < 0.1; * *p* < 0.05; ** *p* < 0.01; *** *p* < 0.001.

**Table 4:** Linear regressions of exposure to maternal distress (perinatal and any time) and family strain as predictors of child allergic symptomatology adjusted for known predictors, current mental health and education.

Child Allergy Symptomatology								
Model	1: PD		2: AMD		3: PA		4: AMA	
	Beta	95% CI	Beta	95% CI	Beta	95% CI	Beta	95% CI
M education	−0.02	−0.58–0.40	−0.21	−0.57–0.40	−0.14	−0.56–0.44	−0.01	−0.53–0.45
Tobacco exposure	0.07	−0.21–0.75	0.06	−0.21–0.74	0.07	−0.19–0.79	0.07	−0.18–0.79
Prenatal antibiotics	0.14 **	0.17–1.26	0.14 **	0.19–1.26	0.15 **	0.23–1.34	0.16 **	0.30–1.39
M any allergy	0.22 ***	0.46–1.37	0.20 ***	0.42–1.31	0.21 ***	0.43–1.36	0.22 ***	0.47–1.39
Index child age	−0.04	−0.11–0.05	−0.07	−0.13–0.03	−0.02	−0.10–0.07	−0.03	−0.10–0.06
Depression score	−0.02	−0.09–0.07	−0.05	−0.11–0.05	0.02	−0.07–0.09	0.02	−0.07–0.09
Anxiety score	−0.18 *	−0.16–−0.02	−0.19 **	−0.16–0.02	−0.14 *	−0.14–−0.01	−0.18 **	−0.16–−0.02
Family strain	0.18 **	0.04–0.22	0.19 ***	0.05–0.23	0.15 **	0.03–0.20	0.15 **	0.03–0.20
Perinatal depression	0.20 ***	0.63–2.15	-	-	-	-	-	-
Perinatal anxiety	-	-	-	-	0.02	−0.73–1.10		
Any depression	-		0.25 ***	0.70–1.80	-	-	-	-
Any anxiety	-		-	-	-	-	0.14 **	0.15–1.20

CI, confidence interval; PD, perinatal depression; AMD, any maternal depression; PA, perinatal anxiety; AMA, any maternal anxiety; M, maternal. * *p* < 0.05; ** *p* < 0.01; *** *p* < 0.001.
